# Sleep, Circadian Rhythms and Cognitive Function: Translational and Real-World Perspectives

**DOI:** 10.3390/brainsci16050460

**Published:** 2026-04-25

**Authors:** Maria Comas

**Affiliations:** 1 Bioaraba Health Research Institute, Neuroscience, Sleep Disorders, Isabel Orbe s/n, 01002 Vitoria-Gasteiz, Spain; comassoberats@gmail.com; 2 IKERBASQUE, Basque Foundation for Science, María Díaz de Haro 3, 48013 Bilbao, Spain; 3 Centre for Integrated Research and Understanding of Sleep (CIRUS), Woolcock Institute of Medical Research, 2 Innovation Rd, Macquarie Park, NSW 2113, Australia

## 1. Introduction

Sleep is a fundamental biological process with broad implications for physical recovery, emotional regulation, and cognitive functioning [1]. Over recent years, growing attention has been focused on the dynamic interplay between sleep, circadian rhythms, and cognition across both clinical and applied contexts [2]. Importantly, this field has expanded well beyond the laboratory, increasingly incorporating real-world settings, ecological assessments, and translational perspectives [3,4]. Despite these advances, major challenges remain in understanding how sleep and circadian processes interact with behavior, performance, subjective experience, and long-term brain resilience [5,6].

This Special Issue, “Sleep, Circadian Rhythms and Cognitive Function”, brings together eight contributions that collectively capture the multifaceted nature of sleep and its impact on cognition. The included articles span systematic and narrative reviews, as well as experimental and real-world studies, addressing sleep in athletic performance, public awareness of circadian-related health topics, real-world sleep variability, insomnia and depressive symptoms, neurobehavioral functioning in attention-deficit/hyperactivity disorder (ADHD), sleep-stage timing under naturalistic conditions, and the emerging relationship between sleep and cognitive reserve. Importantly, the issue also incorporates mechanistic work examining how targeted memory reactivation during sleep modulates cortical connectivity and supports memory consolidation, thereby linking physiological processes with cognitive outcomes. Taken together, these contributions show that variability in sleep and its consequences emerges from the interaction of physiological mechanisms, psychological factors, environmental constraints, and individual differences, with circadian organization acting as a key temporal framework shaping these processes.

## 2. Clinical, Mechanistic and Real-World Perspectives

A first relevant theme emerging from this Special Issue concerns the role of sleep in real-world performance and recovery. In their systematic review, Ochoa-Lácar et al. (Contribution 1) synthesize the evidence on how sleep affects recovery and performance in basketball, highlighting sleep as a key factor in physiological and psychological restoration, injury prevention, and performance optimization. Importantly, their review also emphasizes the contribution of circadian disruption, travel, and training load, showing that sleep-related processes in sport cannot be reduced to sleep duration alone, but should also be interpreted in relation to circadian timing and alignment with training and competition schedules. Moreover, the authors highlight the bidirectional relationship between sleep and training demands, whereby high workloads may impair subsequent sleep and further compromise recovery and performance. This perspective aligns with previous research highlighting the importance of sleep for athletic functioning, while also indicating the complexity of its relationship with performance outcomes [7]. Thus, sleep loss has been consistently associated with impairments in cognitive function and mood but its effects on physical performance appear to be more variable and context dependent. In this sense, the review offers a useful framework for understanding sleep as a multidimensional determinant of performance in ecologically valid settings.

This real-world perspective is further extended by the study by Power et al. (Contribution 2), who examined sleep patterns across a competitive season in a semi-professional female basketball team. Their findings show that sleep fluctuates considerably depending on training and match schedules, with particularly unfavorable patterns observed on training nights before games. These patterns were characterized by reduced sleep duration driven by earlier wake times and, following competition, by later sleep onset and poorer perceived sleep quality. This contribution is especially valuable because it illustrates how average sleep indicators may conceal substantial night-to-night variability, a pattern also reported in elite athletes exposed to early training schedules, where early start times constrain sleep opportunity [8]. Together with Contribution 1, it reinforces the notion that the contextual organization of daily demands, as well as circadian scheduling constraints, play a critical role in recovery and performance.

A second important theme in the Special Issue concerns circadian rhythms, environmental influences, and public awareness. In this regard, Di Simone et al. (Contribution 3) provide an original infodemiological perspective by exploring population-level interest in insomnia, light exposure, metabolism, and circadian-related concepts using Google Trends. Their findings reveal a marked imbalance in public attention. Topics such as insomnia and light exposure attract substantial interest, whereas circadian-related terms remain consistently underrepresented in online searches. This discrepancy suggests that, although strong scientific evidence links circadian disruption to sleep, metabolic health, and overall functioning, these processes are not yet fully integrated into public awareness, despite their central role in structuring daily physiology and cognitive performance across the 24 h cycle.

This gap aligns with the concept of social jetlag, which describes the misalignment between biological and social time, typically quantified as the difference in sleep timing between workdays and free days [9], and highlights the broader disconnect between modern lifestyles and circadian regulation. From a translational perspective, this contribution is particularly relevant because it illustrates how population-level data can be used to identify communication gaps between scientific knowledge and public understanding. In a social context increasingly characterized by artificial light exposure, irregular schedules, and circadian misalignment, improving the visibility and accessibility of circadian science represents a key challenge for prevention and public health.

The interaction between subjective experience, objective sleep measures, and mental health outcomes is another major theme addressed in this Special Issue. In Contribution 4, Comas et al. (Contribution 4) investigated the relationship between anxiety, subjective and objective sleep, chronotype, circadian rhythms, and depressive symptoms in individuals with insomnia disorder. Their findings indicate that subjective measures, particularly anxiety and self-reported sleep- and circadian-related variables, are more strongly associated with depressive symptoms than objective markers such as polysomnographic indices or dim light melatonin onset. Notably, circadian preference, but not objective circadian phase markers, was related to depressive symptoms, pointing to a dissociation between subjective and physiological indices of circadian functioning and highlighting the need to distinguish between perceived timing, biological phase, and their alignment with daily demands. Evidence from circadian research indicates that such misalignment may not be captured by subjective measures alone, as alterations in phase angle can occur independently of chronotype [10]. However, the present findings suggest that objective circadian markers such as dim light melatonin onset do not, in themselves, explain clinical severity, but rather gain relevance when interpreted in relation to sleep timing and behavioral context. This perspective emphasizes that the clinical relevance of circadian phase lies not in its absolute timing, but in its alignment with sleep behavior and daily demands. These findings are consistent with cognitive models of insomnia, which emphasize the role of maladaptive beliefs, attentional biases, and emotional processes in shaping sleep perception and associated outcomes [11]. Overall, this contribution challenges overly reductionist interpretations of sleep pathology and highlights the need to integrate subjective experience, circadian physiology, and behavioral context in both research and clinical practice.

A related but complementary mechanistic perspective is offered by Gruber et al. (Contribution 5), whose systematic review examines the associations between homeostatic and circadian sleep processes and neurobehavioral functioning in individuals with Attention-Deficit/Hyperactivity Disorder (ADHD). Their review supports the idea that perturbations in Process S and Process C are linked to deficits in sustained attention, inhibitory control, and working memory, while also highlighting the importance of their interaction in shaping cognitive performance through their effects on temporal organization, alertness regulation, and alignment with task demands. In particular, factors such as misalignment between circadian preference and time of day, as well as increased daytime sleepiness, appear to play critical roles in neurobehavioral outcomes. At the same time, the authors emphasize that the current literature is limited by substantial methodological weaknesses, including insufficient control of chronotype, time of day, phase angle, and daytime sleepiness. These findings are grounded in the two-process model of sleep regulation, which conceptualizes sleep–wake regulation as the result of the interaction between a homeostatic process (Process S) and a circadian process (Process C), whose effects are dynamically and continuously integrated [12]. Overall, this contribution provides a structured physiological framework for understanding how sleep and circadian regulation influence neurobehavioral functioning, while also identifying key gaps that likely extend beyond ADHD research.

Importantly, this mechanistic perspective is further expanded by the experimental work of Santamaria et al. (Contribution 6), who investigated how targeted memory reactivation during sleep modulates cortical connectivity. Their findings show that auditory cueing during slow-wave sleep induces frequency-specific changes in functional connectivity, particularly within theta-band activity, with increased coupling between frontal and motor-related regions. Crucially, these connectivity patterns not only differentiate between wake and sleep states but also allow discrimination between reactivated memory representations, providing a potential neural marker of memory reactivation. Moreover, the study demonstrates that sleep-related connectivity dynamics are associated with subsequent behavioral performance, linking neural reactivation processes with consolidation outcomes. These findings align with the active system consolidation framework, according to which memory consolidation during sleep is driven by the repeated reactivation of newly encoded representations in the hippocampus and their gradual redistribution to neocortical long-term stores [6]. Within this framework, coordinated oscillatory activity during slow-wave sleep supports large-scale network reorganization, providing a mechanistic basis for the observed connectivity changes. This study therefore provides direct evidence linking sleep-dependent network reorganization to cognitive outcomes and offers a mechanistic bridge between sleep physiology and memory consolidation. In doing so, it complements the broader frameworks outlined in Contribution 5 and highlights the potential of sleep-based interventions to modulate memory processes.

The importance of sleep architecture and temporal organization is addressed in Contribution 7 by von Gall et al. (Contribution 7), who analyzed the timing of deep and Rapid eye movement (REM) sleep under real-life conditions using wearable-based sleep staging in healthy young adults. Their study shows that the temporal coupling between sleep stages and total sleep, reflecting the interaction between homeostatic pressure and circadian phase-dependent organization of sleep throughout the night, is associated with markers of sleep quality, particularly REM sleep consolidation. Notably, workday constraints appear to interfere more strongly with the alignment of deep sleep than with REM sleep, indicating that social schedules may disproportionately affect specific components of sleep architecture. These findings further support the idea that circadian timing not only determines when sleep occurs, but also how its internal structure unfolds across the night. This aligns with recent work linking sleep-stage organization to cognitive functioning and daytime performance [4]. This contribution expands the scope of the Special Issue by showing that sleep timing is not only about when sleep starts and ends, but also about how sleep stages are dynamically organized within the night and how this organization relates to daily functioning and sleep quality.

Finally, the Special Issue opens an especially promising conceptual avenue through the contribution of Balsamo et al. (Contribution 8), who review the complex and still underexplored relationship between sleep and cognitive reserve. Their narrative review highlights a bidirectional perspective. Sleep may act as a builder of cognitive reserve, whereas cognitive reserve may attenuate the negative cognitive effects of poor or disrupted sleep. This perspective is grounded in the concept of cognitive reserve, which explains inter-individual differences in resilience to brain pathology and reflects the ability to optimize or maintain cognitive performance through more efficient or flexible use of brain networks [13]. Importantly, the authors also emphasize that empirical evidence directly linking sleep and reserve remains limited, pointing to a significant gap in the literature. This idea is particularly relevant for understanding why individuals differ in their cognitive and functional responses to sleep disruption. By integrating sleep research with reserve theory, this contribution broadens the Special Issue beyond immediate performance or symptom outcomes and points toward long-term brain resilience as an emerging area of interest.

## 3. Conclusions

The eight contributions included in this Special Issue illustrate the extent and richness of current research on sleep, circadian rhythms, and cognitive function. Rather than supporting a single explanatory framework, they converge on the need for an integrative perspective in which sleep and circadian processes are understood as interacting and mutually shaping systems that influence cognitive and behavioral outcomes. Across athletic, clinical, behavioral, and mechanistic domains, the studies consistently show that cognitive functioning is not determined by isolated factors, but by the dynamic interplay between sleep regulation, circadian timing, psychological state, and environmental demands.

Several broader messages emerge from these contributions. First, both sleep and circadian processes play a central role in real-world functioning, particularly in contexts requiring sustained performance, adaptation, and recovery. Second, circadian factors remain comparatively underrepresented in public awareness and, in some cases, insufficiently accounted for in research design, despite their fundamental role in shaping physiological and cognitive processes. In this regard, increasing attention should be given not only to circadian timing, but also to circadian alignment and misalignment with environmental and social demands, as well as to the robustness and stability of circadian rhythms, as key determinants of cognitive and behavioral outcomes. Third, the interaction between subjective experience and objective physiological measures represents a critical dimension for understanding individual variability, especially in clinical conditions such as insomnia. Fourth, mechanistic frameworks integrating homeostatic and circadian regulation continue to offer strong explanatory potential, but require more consistent and rigorous application across study designs. Finally, the emerging link between sleep, circadian organization, and cognitive reserve opens a particularly relevant translational perspective, suggesting that these processes may contribute not only to short-term cognitive performance but also to long-term resilience across the lifespan.

Collectively, the contributions in this Special Issue support a shift away from viewing sleep or circadian rhythms in isolation, toward an integrated framework in which their interaction is central to understanding cognitive functioning. In this context, future research should move beyond a primary focus on sleep alone and more systematically incorporate circadian timing, alignment, and the robustness of circadian rhythms, as these dimensions critically shape cognitive and behavioral outcomes in real-world settings. Advancing in this direction will require multimodal, longitudinal, and ecologically grounded approaches capable of capturing sleep–circadian dynamics in relation to cognitive functioning. Such an approach may ultimately support the development of more precise and context-sensitive strategies aimed at optimizing sleep–circadian alignment in everyday life and improving cognitive health and performance.
